# MARS: improving multiple circular sequence alignment using refined sequences

**DOI:** 10.1186/s12864-016-3477-5

**Published:** 2017-01-14

**Authors:** Lorraine A. K. Ayad, Solon P. Pissis

**Affiliations:** Department of Informatics, King’s College London, Strand, London, WC2R 2LS UK

**Keywords:** Multiple circular sequence alignment, Circular sequences, *q*-grams, Progressive alignment

## Abstract

**Background:**

A fundamental assumption of all widely-used multiple sequence alignment techniques is that the left- and right-most positions of the input sequences are relevant to the alignment. However, the position where a sequence starts or ends can be totally arbitrary due to a number of reasons: arbitrariness in the linearisation (sequencing) of a circular molecular structure; or inconsistencies introduced into sequence databases due to different linearisation standards. These scenarios are relevant, for instance, in the process of multiple sequence alignment of mitochondrial DNA, viroid, viral or other genomes, which have a circular molecular structure. A solution for these inconsistencies would be to identify a suitable rotation (cyclic shift) for each sequence; these refined sequences may in turn lead to improved multiple sequence alignments using the preferred multiple sequence alignment program.

**Results:**

We present MARS, a new heuristic method for improving Multiple circular sequence Alignment using Refined Sequences. MARS was implemented in the C++ programming language as a program to compute the rotations (cyclic shifts) required to best align a set of input sequences. Experimental results, using real and synthetic data, show that MARS improves the alignments, with respect to standard genetic measures and the inferred maximum-likelihood-based phylogenies, and outperforms state-of-the-art methods both in terms of accuracy and efficiency. Our results show, among others, that the average pairwise distance in the multiple sequence alignment of a dataset of widely-studied mitochondrial DNA sequences is reduced by around 5% when MARS is applied before a multiple sequence alignment is performed.

**Conclusions:**

Analysing multiple sequences simultaneously is fundamental in biological research and multiple sequence alignment has been found to be a popular method for this task. Conventional alignment techniques cannot be used effectively when the position where sequences start is arbitrary. We present here a method, which can be used in conjunction with *any* multiple sequence alignment program, to address this problem effectively and efficiently.

## Background

The one-to-one mapping of a DNA molecule to a sequence of letters suggests that sequence comparison is a prerequisite to virtually all comparative genomic analyses. Due to this, sequence comparison has been used to identify regions of similarity which may be a byproduct of evolutionary, structural, or functional relationships between the sequences under study [1]. Sequence comparison is also useful in fields outside of biology, for example, in pattern recognition [2] or music analysis [3]. Several techniques exist for sequence comparison; alignment techniques consist of either *global* alignment [4, 5] or *local* alignment [6] techniques. Alignment-free techniques also exist; they are based on measures referring to the composition of sequences in terms of their constituent patterns [7]. Pairwise sequence alignment algorithms analyse a pair of sequences, commonly carried out using dynamic-programming techniques [5]; whereas multiple sequence alignment (MSA) involves the simultaneous comparison of three or more sequences (see [8] for a comprehensive review).

Analysing multiple sequences simultaneously is fundamental in biological research and MSA has been found to be a popular method for this task. One main application of MSA is to find conserved patterns within protein sequences [9] and also to infer homology between specific groups of sequences [10]. MSA may also be used in phylogenetic tree reconstruction [11] as well as in protein structure prediction [12].

Using a generalisation of the dynamic-programming technique for pairwise sequence alignments works efficiently for MSA for only up to a few short sequences. Specifically, MSA with the sum-of-pairs score (SP-score) criterion is known to be NP-hard [13]; and, therefore, heuristic techniques are commonly used [14–16], which may not always lead to optimal alignments. As a result, suboptimal alignments may lead to unreliable tree estimation during phylogenetic inference. To this end, several methods aimed to have shown that removing unreliable sites (columns) of an alignment may lead to better results [17].

Several discussions of existing filtering methods provide evidence that the removal of blocks in alignments of sufficient length leads to better phylogenetic trees. These filtering methods take a variety of mathematical and heuristic approaches. Most of the methods are fully automated and they remove entire columns of the alignment. A few of these programs, found in [18, 19], are based on site-wise summary statistics. Several filtering programs, found in [20–24], are based on mathematical models. However, experimental results found in [17] oppose these findings, suggesting that generally, not only do the current alignment filtering methods not lead to better trees, but there also exist many cases where filtering worsened the trees significantly.

Circular molecular structures are present, in abundance, in all domains of life: bacteria, archaea, and eukaryotes; and in viruses. They can be composed of both amino and nucleic acids. Exhaustive reviews can be found in [25] (proteins) and [26] (DNA). The most common examples of such structures in eukaryotes are mitochondrial DNA (mtDNA). mtDNA is generally conserved from parent to offspring and replication of mtDNA occurs frequently in animal cells [27]. This is key in phylogenetic analysis and the study of evolutionary relationships among species [11]. Several other example applications exist including MSA of viroid or viral genomes [28] and MSA of naturally-occurring circular proteins [29].

A fundamental assumption of all widely-used MSA techniques is that the left- and right-most positions of the input sequences are relevant to the alignment. However, the position where a sequence starts (left-most) or ends (right-most) can be totally arbitrary due to a number of reasons: arbitrariness in the linearisation (sequencing) of a circular molecular structure; or inconsistencies introduced into sequence databases due to different linearisation standards. In these cases, existing MSA programs, such as Clustal *Ω* [30], MUSCLE [31], or T-Coffee [16], may produce an MSA with a higher average pairwise distance than the expected one for closely-related sequences. A rather surprising such instance is the published human (NC_001807) and chimpanzee (NC_001643) mtDNA sequences, which do not start in the same genetic region [32]. It may be more relevant to align mtDNA based on gene order [33], however, the tool we present in this paper may be used to align sequences of a broader type. Hence, for a set of input sequences, a solution for these inconsistencies would be to identify a suitable rotation (cyclic shift) for each sequence; the sequences output would in turn produce an MSA with a lower average pairwise distance.

Due to the abundance of circular molecular structures in nature as well as the potential presence of inconsistencies in sequence databases, it becomes evident that multiple circular sequence alignment (MCSA) techniques for analysing such sequences are desirable. Since MCSA is a generalisation of MSA it is easily understood that MCSA with the SP-score criterion is also NP-hard. To this end, a few programs exist which aim to improve MCSA for a set of input sequences. These programs can be used to first obtain the best-aligned rotations, and then realign these rotations by using conventional alignment programs, such as Clustal *Ω*, MUSCLE, or T-Coffee. Note that unlike other filtering programs, these programs do not remove any information from the sequences or from their alignment: they merely refine the sequences by means of rotation.

The problem of finding the optimal (linear) alignment of two circular sequences of length *n* and *m*≤*n* under the edit distance model can be solved in time *O*(*n* *m* log*m*) [34]. The same problem can trivially be solved in time *O*(*n* *m* ^2^) with substitution matrices and affine gap penalty scores [5]. To this end, alignment-free methods have been considered to speed-up the computation [35, 36]. The more general problem of searching for a circular pattern in a text under the edit distance model has also been studied extensively [37], and an average-case optimal algorithm is known [38].

Progressive multiple sequence alignments can be constructed by generalising the pairwise sequence alignment algorithms to profiles, similar to Clustal *Ω* [30]. This generalisation is implemented in Cyclope [39], a program for improving multiple circular sequence alignment. The cubic runtime of the pairwise alignment stage becomes a bottleneck in practical terms. Other fast heuristic methods were also implemented in Cyclope, but they are only based on some (e.g. the first two) sequences from the input dataset.

Another approach to improve MCSA was implemented in CSA [32]; a program that is based on the generalised circular suffix tree construction [40]. The best-aligned rotations are found based on the largest chain of non-repeated blocks that belong to all sequences. Unfortunately, CSA is no longer maintained; it also has the restriction that there can be only up to 32 sequences in the input dataset, and that there must exist a block that occurs in every sequence only once.

BEAR [41] is another program aimed to improve MCSA computation in terms of the inferred maximum-likelihood-based phylogenies. The authors presented two methods; the first extends an approximate circular string matching algorithm for conducting approximate circular dictionary matching. A matrix *M* is outputted from this computation. For a set of *d* input sequences *s*_0_,…,*s*_*d*−1_, *M* holds values *e* and *r* between circular sequences *s*_*i*_ and *s*_*j*_, where *M*[*i*,*j*].*e* holds the edit distance between the two sequences and *M*[*i*,*j*].*r* holds the rotation of sequence *s*_*i*_ which will result in the best alignment of *s*_*i*_ with *s*_*j*_. Agglomerative hierarchical clustering is then used on all values *M*[*i*,*j*].*e*, to find sufficiently good rotations for each sequence cluster. The second method presented is suitable for more divergent sequences. An algorithm for fixed-length approximate string matching is applied to every pair of sequences to find most similar factors of fixed length. These factors can then determine suitable rotations for all input sequences via the same method of agglomerative hierarchical clustering.

**Our contributions.** We design and implement MARS, a new heuristic method for improving Multiple circular sequence Alignment using Refined Sequences. MARS is based on a non-trivial coupling of a state-of-the-art pairwise circular sequence comparison algorithm [35] with the classic progressive alignment paradigm [42]. Experimental results presented here, using real and synthetic data, show that MARS improves the alignments and outperforms state-of-the-art methods both in terms of accuracy and efficiency. Specifically, to support our claims, we analyse these results with respect to standard genetic measures as well as with respect to the inferred maximum-likelihood-based phylogenies. For instance, we show here that the average pairwise distance in the MSA of a dataset of widely-studied mtDNA sequences is reduced by around 5% when MARS is applied before MSA is performed.

### Definitions and notation

We begin with a few definitions, following [43], to allow further understanding. We think of a *string* (or sequence) *x* of *length* *m* as an array *x*[0.. *m*−1] where every *x*[*i*], 0≤*i*<*m*, is a *letter* drawn from some fixed *alphabet* *Σ* of size |*Σ*|=*O*(1). String *ε* denotes the *empty string* which has length 0. Given string *y*, a string *x* is considered a *factor* of *y* if there exist two strings *u* and *v*, such that *y*=*u* *x* *v*. Consider the strings *x*,*y*,*u*, and *v*, such that *y*=*u* *x* *v*. We call *x* a *prefix* of *y* if *u*=*ε*; we call *x* a *suffix* of *y* if *v*=*ε*. When *x* is a factor of *y*, we say that *x* *occurs* in *y*. Each occurrence of *x* can be denoted by a position in *y*. We say that *x* occurs at the *starting position* *i* in *y* when *y*[ *i*.. *i*+*m*−1]=*x*; alternatively we may refer to the *ending position* *i*+*m*−1 of *x* in *y*.

A circular string of length *m* may be informally defined as a standard linear string where the first- and last-occurring letters are wrapped around and positioned next to each other. Considering this definition, the same circular string can be seen as *m* different linear strings, which would all be considered equivalent. Given a string *x* of length *m*, we denote by *x* ^*i*^=*x*[*i*.. *m*−1]*x*[0.. *i*−1], 0<*i*<*m*, the *i*th *rotation* of *x* and *x* ^0^=*x*. By looking at the string *x*=*x* ^0^=baababac; this string has the following rotations: *x* ^1^=aababacb, *x* ^2^=ababacba, *x* ^3^=babacbaa, etc.

Given a string *x* of length *m* and a string *y* of length *n*, the edit distance [44], denoted by *δ*_*E*_(*x*,*y*), is defined as the minimum total cost of operations required to transform string *x* into string *y*. In general, the allowed edit operations are as follows:

- *Insertion*: insert a letter in *y*, not present in *x*; (*ε*,*b*), *b*≠*ε*
- *Deletion*: delete a letter in *y*, present in *x*; (*a*,*ε*), *a*≠*ε*
- *Substitution*: replace a letter in *y* with a letter in *x*; (*a*,*b*), *a*≠*b*,and *a*,*b*≠*ε*.

A *q-gram* is defined as any string of length *q* over alphabet *Σ*. The set of all such *q*-grams is denoted by *Σ* ^*q*^. The *q*-gram profile of a string *x* of length *m* is the vector *G*_*q*_(*x*), where *q*>0, and *G*_*q*_(*x*)[*v*] denotes the total number of occurrences of *q*-gram *v*∈*Σ* ^*q*^ in *x*.

Given strings *x* of length *m* and *y* of length *n*≥*m* and an integer *q*>0, the *q-gram distance* *D*_*q*_(*x*,*y*) is defined as:

1 $$ \sum\limits_{v \in \Sigma^{q}} \left\vert G_{q}(x)[v] - G_{q}(y)[v] \right\vert.  $$

For a given integer parameter *β*≥1, a generalisation of the *q*-gram distance can be defined by partitioning *x* and *y* in *β* *blocks* as evenly as possible, and computing the *q*-gram distance between each pair of blocks, one from *x* and one from *y*. The rationale is to enforce *locality* in the resulting overall distance [35]. Given strings *x* of length *m* and *y* of length *n*≥*m* and integers *β*≥1 and *q*>0, the *β* *-blockwise q-gram distance* *D*_*β*,*q*_(*x*,*y*) is defined as:

2 $${} \sum_{j=0}^{\beta-1}D_{q}\left(\!x\left[\!\frac{jm}{\beta} \ldots \frac{(j+1)m}{\beta}-1\!\right], y\left[\!\frac{jn}{\beta} \ldots \frac{(j+1)n}{\beta}-1\!\right]\right).  $$

We assume that the lengths *m* of *x* and *n* of *y* are both multiples of *β*, so that *x* and *y* are partitioned into *β* blocks, each of size $\frac {m}{\beta }$ and $\frac {n}{\beta }$, respectively.

## Implementation

### Algorithm MARS

We present MARS; a heuristic algorithm for improving MCSA using refined sequences. For a set of *d* *input* sequences *s*_0_,…,*s*_*d*−1_, the task is to *output* an array *R* of size *d* such that *s* ^*R*[*i*]^, for all 0≤*i*<*d*, denotes the set of rotated sequences, which are then input into the preferred MSA algorithm to obtain an improved alignment. MARS is based on a three-stage heuristic approach:

1. Initially a *d*×*d* matrix *M* holding two values *e* and *r* per cell, is computed; where *M*[*i*,*j*].*e* holds the edit distance between sequences $s_{i}^{M[i,j].r}$ and *s*_*j*_. Intuitively, we try to compute the value *r* that minimises *e*, that is, the cyclic edit distance.
2. The neighbour-joining clustering method is carried out on the computed distances to produce a *guide tree*.
3. Finally, progressive sequence alignment using refined sequences is carried out using the sequence ordering in the guide tree.

#### Stage 1. Pairwise cyclic edit distance

In this stage, we make use of a heuristic method for computing the cyclic edit distance between two strings. This method is based on Grossi et al’s alignment-free algorithm [35] for circular sequence comparison, where the *β*-blockwise *q*-gram distance between two circular sequences *x* and *y* is computed. Specifically, the algorithm finds the rotation *r* of *x* such that the *β*-blockwise *q*-gram distance between *x* ^*r*^ and *y* is minimal.

The second step of this stage involves a refinement of the rotation for a pair of sequences, to obtain a more accurate value for *r*. An input parameter $0 < P \leq \frac {\beta }{3}$ is used to create refined sequences of length $3 \times P \times \frac {m}{\beta }$ using *x* ^*r*^ and *y*, where m is the length of *x* ^*r*^. The first refined sequence is ${x^{r}_{0}}{x^{r}_{1}}{x^{r}_{2}}$: ${x^{r}_{0}}$ is a prefix (of *P* out of *β* blocks) of string *x* ^*r*^; ${x^{r}_{1}}$ is a string of the same length as the prefix consisting only of letter *$*∉*Σ*; and ${x^{r}_{2}}$ is a suffix (of *P* out of *β* blocks) of string *x* ^*r*^. The same is done for string *y*, resulting in a refined sequence of the same form *y*_0_ *y*_1_ *y*_2_. Note that large values for *P* would result in long sequences, improving the refinement of the rotation, but slowing down the computation. A score is calculated for all rotations of these two smaller sequences using Needleman-Wunsch [4] or Gotoh’s algorithm [5], making use of substitution matrices for nucleotides or amino acids accordingly. The rotation with the maximum score is identified as the new best-aligned rotation and *r* is updated if required.

The final step of this stage involves computing the edit distance between the new pair of refined sequences. For unit costs, this is done using Myers bit-vector algorithm [45] in time $O\left (\left \lceil {\frac {m}{w}}\right \rceil n\right)$, where *w* is the word size of the machine. For non-unit costs this is computed using the standard dynamic programming solution for edit distance [44] computation in time *O*(*m* *n*). Hence, for a dataset with *d* sequences, a *d*×*d* matrix *M* is populated with the edit distance *e* and rotation *r* for each pair of sequences.

##### **Remark for Stage 1**

The simple cost scheme used in Stage 1 for the pairwise cyclic edit distance is sufficient for computing fast approximate rotations. A more complex (biologically relevant) scoring scheme is used in Stage 3 for refining these initial rotations. A yet more complex scoring scheme, required for the final MSA of the sequences output by MARS, can be carried out later on by using any MSA program, and is therefore beyond the scope of this article.

#### Stage 2. Guide tree

The guide tree is constructed using Saitou and Nei’s neighbour-joining algorithm [46], where a binary tree is produced using the edit distance data from matrix *M*.

#### Stage 3. Progressive alignment

The guide tree is used to determine the ordering of the alignment of the sequences. Three types of alignments may occur:

- Case 1: A sequence with another sequence;
- Case 2: A sequence with a profile;
- Case 3: A profile with another profile;

where a *profile* is an alignment viewed as a sequence by regarding each column as a letter [14]. We also need to extend the alphabet to *Σ* ^′^=*Σ*∪{−} to represent insertions or deletions of letters (gaps). For the rest of this stage, we describe our method using the Needleman-Wunsch algorithm for simplicity although Gotoh’s algorithm is also applicable.

For Case 1, where only two sequences are to be aligned, note that rotation *r* has been previously computed and stored in matrix *M* during Stage 1 of the algorithm. These two sequences are aligned using Needleman-Wunsch algorithm and stored as a new profile made up of the alignment of two individual sequences which now include gaps. In this case, for two sequences *s*_*i*_ and *s*_*j*_, we set *R*[*i*]:=*M*[*i*,*j*].*r* and *R*[*j*]:=0, as the second sequence is left unrotated.

The remaining two cases of alignments are a generalisation of the pairwise circular sequence alignment to profiles. In the alignment of a pair of sequences, matrix *M* provides a reference as to which rotation *r* is required. In the case of a sequence and a profile (Case 2), this may also indirectly be used as we explain below.

As previously seen, when two sequences *s*_*i*_ and *s*_*j*_ are aligned, one sequence *s*_*j*_ remains unrotated. This pair then becomes a profile which we will call *profile A*. Given the same occurs for another pair of sequences, *profile B* is created also with one unrotated sequence, $s_{j^{\prime }}\phantom {\dot {i}\!}$. When *profile A* is aligned with *profile B*, another profile, *profile C* is created. In this case, only the sequences in *profile B* are rotated to be aligned with *profile A*. This results in *s*_*j*_ to be left unrotated in *profile C* where *s*_*j*_ previously occurred in *profile A*. Given a sequence *s*_*k*_ to be aligned with *profile C*, this sequence has a current rotation of 0 as has not yet been aligned with another sequence or a profile. We can identify which rotation is needed to rotate sequence *s*_*k*_ to be aligned with *profile C*, by using the single rotation *M*[ *k*,*j*].*r*.

The same condition applies when aligning two profiles (Case 3). All sequences in *profile B* will need to be rotated to be aligned with *profile A*. However, once a single sequence *s*_*j*_ in *profile A* as well as a single sequence $s_{j^{\prime }}\phantom {\dot {i}\!}$ in *profile B* with *r*=0 have been identified, in this case $s_{j^{{\prime }}}\phantom {\dot {i}\!}$ has already been aligned with other sequences. This means gaps may have been inserted and *M*[*j* ^′^,*j*].*r* will no longer be an accurate rotation. By counting the total number *g* of individual gaps inserted in $s_{j^{{\prime }}}\phantom {\dot {i}\!}$, between positions 0 and the single rotation *M*[*j* ^′^,*j*].*r* of $s_{j^{\prime }}\phantom {\dot {i}\!}$, the new suitable rotation for *profile B* would be *M*[*j* ^′^,*j*].*r*+*g*.

##### **Example**

Consider the following sequences:

*s*_0_: TAGTAGCT

*s*_1_: AAGTAAGCTA

*s*_2_: AAGCCTTTAGT

*s*_3_: AAGTAAGCT

*s*_4_: TTAATATAGCC

Let *profile A* be:

*s*_0_: A - G - C - - TTA - GT

*s*_1_: AAG - C - - TAAAGT

*s*_2_: AAGCC - TTTA - GT

Let *profile B* be:

*s*_3_: A - - - AGTAAG - C - - T

*s*_4_: A - ATA - TA - GCC - TT

*Profile C*:

*s*_0_: A - G - C - - TT - A - - GT

*s*_1_: AAG - C - - TA - A - AGT

*s*_2_: AAGCC - TTT - A - - GT

*s*_3_: AAG - C - - TA - - - AGT

*s*_4_: A - GCC - TTA - ATA - T

By looking at the original set of sequences, it is clear *s*_2_ in *profile A* and *s*_3_ in *profile B* have a rotation of 0. The other sequences have been rotated and aligned with the remaining sequences in their profile. It is also clear from the original sequences that *M*[3,2].*r*=4. When aligning *profile B* with *profile A*, the rotation of *r*=4 is no longer accurate due to gaps inserted in *s*_3_. As *g*=3 gaps have been inserted between positions 0 and *r* of sequence *s*_3_, the final accurate rotation for *profile B* is *M*[ 3,2].*r*+*g*=4+3=7 (see *profile C*). □

In the instance when a sequence is to be aligned with a profile or two profiles are to be aligned, a generalisation of the Needleman-Wunsch algorithm is used, similar to that by [47], to compute the alignment score. *Profile A* will always hold the largest number of sequences, allowing *profile B* with fewer sequences to be rotated.

A frequency matrix *F* is stored, which holds the frequency of the occurrence of each letter in each column in *profile A*. Equation 3 shows the scoring scheme used for each alignment, where *S*[*i*,*j*] holds the alignment score for column *i* in *profile A* and column *j* in *profile B*. *gA* is the cost of inserting a gap into *profile A* and *gB* likewise for *profile B*. Matrix *S* is initialised in the same way as in the Needleman-Wunsch algorithm; and *sim*(*B*[*k*,*j*],*c*) denotes the similarity score between letter *c*∈*Σ* ^′^ and the letter at column *j* of row *k* (representing sequence *s*_*k*_) in *profile B*.

3 $$\begin{array}{@{}rcl@{}} S[\!i,j] &=& \max \left\{ {\begin{array}{l} S[i-1,j-1] + \textit{pScore}(i,j) \\ S[i-1,j] + \textit{gB} \\ S[i,j-1] + \textit{gA} \end{array}} \right.\\ \textit{pScore}(i,j) &=& \sum_{c \in \Sigma'}{}\! \textit{sim}(B[\!k,j],c)\times F[\!c,i] 0 \leq k \!< |B| \end{array} $$

This scoring scheme can be applied naïvely for *profile A* and every rotation of *profile B* to find the maximum score, equating to the best-aligned rotation. However, as information about rotations has already been computed in Stage 1, we may use only some part of *profile B* to further refine these rotations. This refinement is required due to the heuristic computation of all pairwise cyclic edit distances in Stage 1 of the algorithm. To this end, we generalise the second step of Stage 1 to profiles. This step of Stage 1 involves a refinement of the rotation for a pair of sequences via considering only the two ends of each sequence, to create two refined sequences. Similarly here we generalise this idea to refine the rotation for a pair of profiles via considering only the two ends of each profile, to recreate the profiles into profiles with refined sequences. The rotation *r* with the maximum score according to the aforementioned scoring scheme is identified as the best-aligned rotation and array *R* is updated by adding *r* to the current rotation in *R*[*i*], for all *s*_*i*_ in profile *B*.

## Results

MARS was implemented in the C++ programming language as a program to compute the rotations (cyclic shifts) required to best align a set of input sequences. Given a set of *d* sequences in multiFASTA format, the length *ℓ* of the *β* blocks to be used, the length *q* of the *q*-grams, and a real number *P* for the refinement, MARS computes an array *R* according to the algorithm described in the “Implementation” section. There is also a number of optional input parameters related to Gotoh’s algorithm, such as the gap opening and extension penalty scores for pairwise and multiple sequence alignment. A different substitution matrix can be used for scoring nucleotide or amino acid matches and mismatches. The output of MARS is another multiFASTA file consisting of *d* refined sequences, produced using the rotations computed in *R*. The output of MARS can then be used as input to the preferred MSA program, such as Clustal *Ω*, MUSCLE, or T-Coffee.

The implementation is distributed under the GNU General Public License (GPL), and it is available freely at http://github.com/lorrainea/mars. Experimental results were also produced for Cyclope and BEAR to compare their performance against MARS. The experiments were conducted on a computer using an Intel Core i5-4690 CPU at 3.50 GHz under GNU/Linux. All programs were compiled with g++ version 4.8.5 at optimisation level 3 (O3).

### Synthetic data

DNA datasets were simulated using INDELible [48], which produces sequences in a multiFASTA file. A rate for insertions, deletions, and substitutions are defined by the user to vary similarity between the sequences. All datasets used in the experiments are denoted in the form A.B.C, where A represents the number of sequences in the dataset; B the average length of the sequences; and C the percentage of dissimilarity between the sequences. Substitution rates of 5, 20, and 35% were used to produce the datasets under the Jukes and Cantor (JC69) [49] substitution model. The insertion and deletion rates were set to 4 and 6% respectively, relative to a substitution rate of 1.

Nine datasets were simulated to evaluate the accuracy of the proposed method. Each dataset consists of a file with a varying number of sequences, all with an average length of 2500 base pairs (bp). The files in Datasets 1−3 each contain 12 sequences. Those in Datasets 4−6 each contain 25 sequences; and Datasets 7−9 contain 50 sequences. All input datasets referred to in this section are publicly maintained at the MARS website.

For all datasets, we made use of the following values for the mandatory parameters of MARS: *q*=5, *ℓ*=50, and *P*=1.0. Table 1 shows the results for the synthetic datasets made up of three files which each contained 12 sequences (Datasets 1–3). The first column shows results for the original datasets aligned using Clustal *Ω*. All sequences in these datasets were then randomly rotated, denoted in Table 1 by A.B.C.rot. The second column shows the results produced when MARS was first used to refine the sequences in the A.B.C.rot dataset, to find the best-aligned rotations; and then aligned them again using Clustal *Ω*. The third and fourth columns show likewise using MUSCLE to align the sequences. Tables 2 and 3 show the results produced for Datasets 4–6 and 7–9, respectively.

**Table 1:** Standard genetic measures for Datasets 1-3

Program	Clustal *Ω*	MARS+ Clustal *Ω*	MUSCLE	MARS+ MUSCLE
Dataset 1	12.2500.5	12.2500.5.rot	12.2500.5	12.2500.5.rot
Length	2,503	2,503	2,503	2,503
PM Sites	698	698	689	689
Transitions	3,845	3,849	3,804	3,804
Transversions	4,245	4,251	4,205	4,205
Substitutions	12,254	12,264	12,111	12,111
Indels	360	360	388	388
AVPD	191	191	189	189
Dataset 2	12.2500.20	12.2500.20.rot	12.2500.20	12.2500.20.rot
Length	2,662	2,664	2,674	2,674
PM Sites	2,228	2,230	2,155	2,155
Transitions	16,819	16,502	16,171	16,184
Transversions	15,374	15,719	14,422	14,422
Substitutions	47,754	47,799	44,261	44,280
Indels	10,545	10,707	8,817	8,815
AVPD	883	886	804	804
Dataset 3	12.2500.35	12.2500.35.rot	12.2500.35	12.2500.35.rot
Length	2,526	2,528	2,528	2,528
PM Sites	2,062	2,070	2,045	2,045
Transitions	18,385	18,167	18,362	18,362
Transversions	17,642	17,728	17,316	17,316
Substitutions	54,573	54,533	53,807	53,807
Indels	2,403	2,575	2,253	2,253
AVPD	863	865	849	849

**Table 2:** Standard genetic measures for Datasets 4–6

Program	Clustal *Ω*	MARS+ Clustal *Ω*	MUSCLE	MARS+ MUSCLE
Dataset 4	25.2500.5	25.2500.5.rot	25.2500.5	25.2500.5.rot
Length	2,515	2,515	2,515	2,515
PM Sites	1,243	1,238	1,230	1,230
Transitions	20,438	20,422	20,353	20,353
Transversions	20,672	20,587	20,498	20,498
Substitutions	61,780	61,523	61,289	61,289
Indels	2,582	1,932	1,842	1,842
AVPD	214	211	210	210
Dataset 5	25.2500.20	25.2500.20.rot	25.2500.20	25.2500.20.rot
Length	2,600	2,595	2,590	2,591
PM Sites	2,585	2,577	2,572	2,572
Transitions	105,738	105,596	106,070	106,256
Transversions	104,778	104,451	103,335	103,238
Substitutions	313,329	312,311	309,953	310,056
Indels	20,524	20,658	13,678	13,784
AVPD	1,112	1,109	1,078	1,079
Dataset 6	25.2500.35	25.2500.35.rot	25.2500.35	25.2500.35.rot
Length	2,726	2,751	2,722	2,716
PM Sites	2,700	2,727	2,684	2,679
Transitions	101,801	102,471	104,001	103,796
Transversions	104,993	104,632	100,595	101,078
Substitutions	310,597	311,468	304,100	304,481
Indels	47,080	58,288	35,956	35,110
AVPD	1,192	1,232	1,133	1,131

**Table 3:** Standard genetic measures for Datasets 7–9

Program	Clustal *Ω*	MARS+ Clustal *Ω*	MUSCLE	MARS+ MUSCLE
Dataset 7	50.2500.5	50.2500.5.rot	50.2500.5	50.2500.5.rot
Length	2,524	2,524	2,524	2,524
PM Sites	1,875	1,882	1,861	1,861
Transitions	86,781	87,190	86,628	86,628
Transversions	91,334	91,584	91,040	91,040
Substitutions	262,804	263,687	261,248	261,248
Indels	11,531	10,771	8,231	8,231
AVPD	223	224	219	219
Dataset 8	50.2500.20	50.2500.20.rot	50.2500.20	50.2500.20.rot
Length	2,576	2,580	2,582	2,582
PM Sites	2,568	2,573	2,575	2,575
Transitions	284,302	284,667	282,638	282,670
Transversions	283,651	284,673	279,451	279,462
Substitutions	852,738	855,055	842,564	842,672
Indels	39,273	45,769	33,371	33,369
AVPD	728	735	715	715
Dataset 9	50.2500.35	50.2500.35.rot	50.2500.35	50.2500.35.rot
Length	2,675	2,697	2,679	2,667
PM Sites	2,675	2,696	2,678	2,666
Transitions	424,910	423,592	426,230	426,063
Transversions	431,453	428,874	423,113	422,916
Substitutions	1,282,515	1,278,286	1,267,683	1,267,699
Indels	92,060	97,398	73,890	72,718
AVPD	1,122	1,123	1,095	1,094

To evaluate the accuracy of MARS seven standard genetic measures were used: the length of the MSA; the number of polymorphic sites (PM sites); the number of transitions and transversions; substitutions, insertions, and deletions were also counted; as well as the average distance between each pair of sequences in the dataset (AVPD).

#### **Remark for accuracy**

The use of standard genetic measures to test the accuracy of MARS with synthetic data is sufficient. This is due to the fact that the main purpose of this test is not to check whether we obtain an MSA that is biologically relevant. The ultimate task here was to show that when MARS is applied on the A.B.C.rot datasets before MSA is performed we obtain MSAs whose standard genetic measures are similar or even identical to the MSAs of the A.B.C datasets (and this cannot occur by chance) when using the *same* MSA program.

The results show indeed that MARS performs extremely well for all datasets. This can be seen through the high similarity between the measurements for the original and the refined datasets. Notice that, in particular with MUSCLE, we obtain an *identical or less* average pairwise distance in 8 out of 9 cases between the original and the refined datasets produced by using MARS, despite the fact that we had first randomly rotated all sequences (compare the A.B.C to the A.B.C.rot columns).

RAxML [50], a maximum-likelihood-based program for phylogenetic analyses, was used to identify the similarity between the phylogenetic trees inferred using the original and refined datasets. A comparison with respect to the phylogenetic trees obtained using MUSCLE and RAxML was made between the alignment of the original datasets and that of the datasets produced by refining the A.B.C.rot datasets using MARS, BEAR, and Cyclope. The Robinson–Foulds (RF) metric was used to measure the distance between each pair of trees. The same parameter values were used for MARS: *q*=5, *ℓ*=50, and *P*=1.0. The fixed-length approximate string matching method with parameter values *w*=40 and *k*=25 under the edit distance model, were used for BEAR, where *w* is the factor length used and *k* is the maximum distance allowed. Parameter *v* was used for Cyclope to compute, similar to MARS, a tree-guided alignment.

Table 4 shows that the *relative* RF distance between the original datasets and those refined with MARS is 0 in all cases, showing that MARS has been able to identify the best-aligned rotations, with respect to the inferred trees, for all nine datasets, outperforming BEAR and Cyclope, for which we obtain non-zero values in some cases.

**Table 4:** Relative RF distance between trees obtained with original and refined datasets

Dataset	BEAR	Cyclope	MARS
12.2500.5	0.000	0.000	0.000
12.2500.20	0.000	0.000	0.000
12.2500.35	0.000	0.000	0.000
25.2500.5	0.000	0.000	0.000
25.2500.20	0.000	0.000	0.000
25.2500.35	0.000	**0.045**	0.000
50.2500.5	**0.021**	0.000	0.000
50.2500.20	0.000	0.000	0.000
50.2500.35	0.000	0.000	0.000

Non-zero values shown in bold

### Real data

In this section we present the results for three datasets used to evaluate the effectiveness of MARS with real data. The first dataset (Mammals) includes 12 mtDNA sequences of mammals, the second dataset (Primates) includes 16 mtDNA sequences of primates, and the last one (Viroids) includes 18 viroid RNA sequences. All input datasets referred to in this section are publicly maintained at the MARS website. The average sequence length for Mammals is 16,777 bp, for Primates is 16,581 bp, and for Viroids is 363 bp.

Table 5 shows the results from the original alignments and the alignments produced after refining these datasets with MARS. It is evident that using MARS produces a significantly better alignment for these real datasets, which can specifically be seen through the results produced by aligning with MUSCLE. For instance, the average pairwise distance in the MSA of Primates is reduced by around 5% when MARS is applied before MSA is performed with MUSCLE.

**Table 5:** Standard genetic measures for real data

Program	Clustal *Ω*	MARS+ Clustal *Ω*	MUSCLE	MARS+ MUSCLE
Mammals				
Length	19,452	18,829	19,784	19,180
PM Sites	12,913	12,265	13,076	12,454
Transitions	135,380	137,589	135,794	137,835
Transversions	81,945	84,188	76,894	78,067
Substitutions	295,684	302,331	282,608	286,747
Indels	82,494	59,348	91,164	71,042
AVPD	5729	5479	5663	5421
Primates				
Length	18,176	17,568	18,189	17,669
PM Sites	11,086	10,450	11,023	10,454
Transitions	259,921	261,995	262,179	264,245
Transversions	100,708	102,336	95,403	96,010
Substitutions	439,929	445,252	429,532	432,993
Indels	80,851	52,727	82,117	55,525
AVPD	4339	4149	4263	4070
Viroids				
Length	566	498	486	476
PM Sites	555	484	475	459
Transitions	7567	7485	9338	9101
Transversions	5837	5998	5491	5393
Substitutions	19,436	19,291	20,828	20,374
Indels	19,003	18,383	14,323	13,491
AVPD	251	246	229	221

Since time-accuracy is a standard trade-off of heuristic methods, in order to evaluate the time performance of the programs, we compared the resulting MSA along with the time taken to produce it using BEAR, Cyclope, and MARS with MUSCLE. Parameter values *h*=100 and *k*=60 were used to measure accuracy for the Mammals and Primates datasets for BEAR; *w*=40 and *k*=25 were used for the Viroids dataset. Parameter *v* was used for Cyclope to compute a tree-guided alignment. The following parameter values were used to test the Mammals and Primates datasets for MARS: *q*=5, *ℓ*=100, and *P*=2.0; *q*=4, *ℓ*=25, and *P*=1.0 were used to test the Viroids dataset.

Table 6 shows the time taken to execute the datasets; for the sake of succinctness, Table 6 only presents the average pairwise distance measure for the quality of the MSAs. The results show that MARS has the best time-accuracy performance: BEAR is the fastest program for two of the three datasets, but produces very low-quality MSAs; Cyclope is very slow but produces much better MSAs than BEAR; and MARS produces better MSAs than both BEAR and Cyclope and is more than four times faster than Cyclope.

**Table 6:** Elapsed-time comparison using real data

Program	BEAR		Cyclope		MARS	
Dataset	AVPD	Time (s)	AVPD	Time (s)	AVPD	Time (s)
Mammals	5517	262.96	5422	1367.17	5421	333.50
Primates	4167	465.17	4080	2179.68	4070	463.25
Viroids	232	0.30	223	1.44	221	0.82

A common reliability measure of MSAs is the computation of the transitive consistency score (TCS) [51]. The TCS has been shown to outperform existing programs used to identify structurally correct portions of an MSA, as well as programs which aim to improve phylogenetic tree reconstruction [8]. BEAR, Cyclope, and MARS were used to identify the best rotations for the sequences in the Viroids dataset; the output of each, as well as the unrotated dataset was then aligned using MUSCLE. The following TCS was computed for the Viroids dataset when unrotated: 260, as well as when rotated with BEAR, Cyclope, and MARS, respectively: 249, 271, and 293. The same was done using Clustal *Ω* to align the output sequences, with a TCS of 249 for the unrotated dataset. The following scores were computed for the rotated dataset in the respective order: 233, 244, and 269. These results show that when using two different MSA programs, MARS obtains a higher TCS than the unrotated dataset in both cases, outperforming BEAR and Cyclope, which do not always obtain a higher TCS compared to that of the unrotated dataset.

## Conclusions

A fundamental assumption of all widely-used MSA techniques is that the left- and right-most positions of the input sequences are relevant to the alignment. This is not always the case in the process of MSA of mtDNA, viroid, viral or other genomes, which have a circular molecular structure.

We presented MARS, a new heuristic method for improving Multiple circular sequence Alignment using Refined Sequences. Experimental results, using real and synthetic data, show that MARS improves the alignments, with respect to standard genetic measures and the inferred maximum-likelihood-based phylogenies, and outperforms state-of-the-art methods both in terms of accuracy and efficiency. We anticipate that further development of MARS would be desirable upon dissemination. Our immediate target is to employ low-level code optimisation and thread-level parallelism to further enhance the performance of MARS. A web-service for improving multiple circular sequence alignment based on MARS is already under way.

## Availability and requirements

**Project name:** MARS **Project home page:** https://github.com/lorrainea/mars **Operating system:** GNU/Linux **Programming language:** C++ **Other requirements:** N/A **License:** GNU GPL

## Abbreviations

AVPD: Average pairwise distance
bp: Base pairs
MARS: Multiple sequence alignment using refined sequences
MCSA: Multiple circular sequence alignment
MSA: Multiple sequence alignment
mtDNA: Mitochondrial DNA
PM sites: Polymorphic sites
RF: Robinson-Foulds
SP-score: Sum-of-pairs score
TCS: Transitive consistency score
